# The applicability of the Eating Disorder Inventory in pregnancy

**DOI:** 10.1007/s40519-021-01197-2

**Published:** 2021-05-07

**Authors:** Szilvia Dukay-Szabó, Dávid Simon, Márta Varga, Orsolya Koller, Zoltán Pataki, János Rigó, Ferenc Túry

**Affiliations:** 1 Institute of Behavioural Sciences, Doctoral School of Mental Health Sciences, Semmelweis University, Nagyvárad tér 4, 1089 Budapest, Hungary; 2 Faculty of Social Sciences, Eötvös Loránd University, Budapest, Hungary; 3 Faculty of Humanities and Social Sciences, Pázmány Péter Catholic University, Budapest, Hungary; 4 Institute of Behavioural Sciences, Semmelweis University, Budapest, Hungary; 5 Uzsoki Hospital, Budapest, Hungary; 6 Department of Obstetrics and Gynaecology, Semmelweis University, Budapest, Hungary; 7 Department of Clinical Studies in Obstetrics and Gynaecology, Semmelweis University, Budapest, Hungary

**Keywords:** Eating disorders, Pregnancy, Eating disorder inventory, Validity, Reliability

## Abstract

**Purpose:**

The aim of our study was validating Eating Disorder Inventory (EDI) among pregnant women, who are vulnerable to eating disorders (EDs).

**Methods:**

In 2012–2013, 1146 women (aged 18–47 years) completed a questionnaire including EDI during the first 3 days after delivery. We checked factorial validity of three diagnostic subscales of EDI with confirmative factor analysis and internal validity by Cronbach’s alpha and item-total correlation. We also tested discriminative validity by comparing average of the three subscale of EDI in case of ED and non-ED groups.

**Results:**

When applying the EDI to pregnant women, it seems necessary to exclude five items on three diagnostic subscales: on the Drive for Thinness subscale, 4 items remain (out of 7); on the Bulimia subscale, 6 items remain (out of 7); the Body Dissatisfaction subscale decreases from 9 to 8 items. Cronbach’s alpha and item-total correlation values meet the requirements defined by Garner et al. The internal consistency of the EDI has proved to be appropriate, indicating that it is a reliable screening tool.

**Conclusions:**

Thinking, attitudes, and behaviors connected to eating, along with the relation to altering body weight change during pregnancy. Vomiting usually accompanies pregnancy; body weight gain within wide limits is also regarded as normal during pregnancy. These behaviors and changes are not feasible to use for measuring ED symptoms. These aspects cannot be neglected when screening eating disorders in pregnant women.

**Level of evidence:**

Level IV evidence obtained from multiple time series with or without an intervention.

## Introduction

Eating disorders (EDs) have become increasingly frequent since the middle of the last century [1]. In addition to individual factors, the social environment plays a significant part in their occurrence. The spectrum of eating disorders has broadened, and new conditions are constantly appearing. Together with obesity, eating disorders are now a major issue in public health [2]. Since the early recognition of a disorder is one of the most important criteria for a good prognosis [3], there is a great need for easy-to-use screening methods.

EDs connected with pregnancy have a special importance for at least three reasons. First, the onset of the EDs occurs typically in adolescence or in young adulthood among women at a critical phase of women’s reproductive life [4], and it appears among 7.5–11.5% of pregnant women) [5, 6]. Second, EDs regularly involve a lack of insight or the hiding of somatic symptoms, and psychological factors make diagnosis more difficult in case of pregnancy [7]. Third, among women in these age groups, psychosomatic disorders can have numerous consequences for pregnancy, its outcome, the development of the fetus, and the postpartum period [6, 8–11]. In addition to the classic eating disorders (anorexia nervosa, bulimia nervosa, and binge eating disorder), a condition specifically associated with pregnancy: pregorexia, which is used to describe women who try to reduce weight gain by dieting and exercising to avoid obesity and retain their slender shape has also been described [12]. Despite the presence of this phenomenon, however, the problem in the developed world is not primarily malnutrition, but excessive weight gain and obesity. [2, 12]. Accordingly, screening for EDs among women in pregnancy is of crucial importance.

Physiological changes and events of the pregnancy can cause problems and biases in screening pregnant women for EDs [13]. Although there are general screening tools for mental disorders that have already been validated among pregnant women [14], and a Delphi consensus study investigated possible items recognizing symptoms of disordered eating [15], to our knowledge, this has not been done for any ED specific measure. This article focuses on the three symptom-related diagnostic EDI subscales for which physiological changes during pregnancy are most likely to cause interference (Drive for Thinness, Body Dissatisfaction, and Bulimia subscales).

The EDI was developed by Garner and colleagues in 1983 [16], and according to recent review studies [17–19], it is one of the most widely used instruments both for the assessment of symptoms and evaluation of treatment effect in clinical practice and for research. Garner et al. validated EDI by checking internal consistency (by Cronbach alpha), criterion-related validity (comparing ED and non-ED groups), and convergent and discriminant validity (based on correlation with other scales) [16]. A meta-analysis conducted by Gleaves et al. [20] proved high reliability of EDI by analyzing published Cronbach alphas. More recently, there have been detailed analyses of the dimensionality [21, 22] and discriminant validity [23] of EDI. Evaluation of EDI has been repeated successfully in different countries (e.g., Austria [24], Hong Kong [25], Hungary [26], and Sweden [27]) and in different subpopulations (e.g., adolescents [28] and athletes [29]), but the evaluation has not been repeated among pregnant women.

The aim of our paper is to validate the widely used Eating Disorder Inventory (EDI) among pregnant women, a specific population which is vulnerable to eating disorders. The topicality and significance of the issue is yielded by the fact that among young women, the incidence of eating disorders is high and it also appears among pregnant women. Since pregnancy is associated with both physical and mental changes, and some of these changes manifest themselves in forms similar to eating disorder symptoms, the validation of the measure should be repeated in the pregnant population.

## Method

### Participants

We conducted a cross-sectional survey in the 1st Department of Obstetrics and Gynaecology, Semmelweis University, Budapest, between May 2012 and April 2013. All mothers included in the research had had full-term pregnancies, their infants were healthy, and they were able to read and write in Hungarian. Mothers of stillbirths or of infants placed in the intensive-care unit were excluded. Participation was voluntary, and all the participants signed an informed consent. Nurses employed in the puerperal department distributed and collected the survey materials, which increased compliance. The women received the questionnaire immediately after delivery and completed it no later than 3 days, while they were in the maternity ward.

Ethical permission was given by the Semmelweis University Regional and Institutional Committee of Science and Research Ethics (Nr 12631/2012/EKU. (212/PI/12.)).

The questionnaire was filled by 1146 women altogether (the overall response rate was 88.2%). The mean age of the respondents was 32.1 years (sd = 5.16 with a range of 18–47 years). Table 1 shows the distribution of the sample.

**Table 1:** Demographic and anthropometric data of the respondents

	*N*	%
Total	1146	100.0
Age groups		
Below 26 years	109	9.5
26–30 years	300	26.2
31–35 years	403	35.2
Above 35 years	315	27.5
No data	19	1.7
Mean age (years 〈SD〉; [range])	32.1 years 〈5.16〉 [18–47]	
Qualification		
Primary education	69	6.0
Secondary education with vocational training	86	7.5
Secondary education with final exams	367	32.0
College/university degree	621	54.2
No data	3	0.3
Type of settlement		
Budapest	574	50.1
City or town	395	34.5
Village	169	17.0
No data	8	0.7
Before pregnancy		
Underweight (BMI < 18.5)	81	7.1
Normal weight (18.5 ≤ BMI < 25)	729	63.6
Overweight (25 ≤ BMI < 30)	211	18.4
Obese (BMI ≥ 30)	103	9.0
No data	22	1.9
Mean BMI (kg/m^2^ 〈SD〉 ; [range])	23.41 kg/m^2^ 〈4.63〉 [15.4–47.8]	

### Measures

Our retrospective questionnaire contained items about demographic and anthropometric data, feelings and attitudes related to body weight, body shape, and their changes during pregnancy, history of former pregnancies, details of the last pregnancy, lifestyle before and during pregnancy, as well as detailed questions on eating habits and the respondent’s relation to eating and her own body in the past 3–6 months.

Identification of EDs was based on self-reporting. We defined the ED group according to whether respondents reported experiencing any somatic or psychological symptoms of EDs: we asked the women in the study whether they had ever had an ED in their lifetime, 1 year before pregnancy or during pregnancy. The ED group comprised women who reported that they had an ED during their pregnancy. Among the respondents, 84 (7.3%) had been affected by ED at some time during their lives, based on self-report, of whom 58 (5.1%) stated that they had had EDs sometime in the past (the ex-ED group) and 26 (2.2%) were currently experiencing ED (the ED group), while 1054 (92%) were stated that they had not suffered from ED at all (the non-ED group). Eight mothers did not respond to this question. (See Table 2.)

**Table 2:** Distribution of self-reported eating disorders in the sample

	*N*	%
**Total**	**1146**	**100.00**
**Self-reported eating disorder during the last pregnancy or in the past altogether**	**84**	**7.33**

*AN*	*19*	*1.66*
*BN*	*13*	*1.13*
*EDNOS*	*46*	*4.01*
* No data about the type*	*6*	*0.52*

People having had an eating disorder in the past	58	5.06
Having had an eating disorder in the past and in connection with pregnancy	2	0.17
Having had an eating disorder in connection with pregnancy but not in the past	24	2.09

**No data by self-report**	**8**	**0.70**
**Not reported any eating disorders either in the last pregnancy or in the past**	**1054**	**91.97**

Bold values are as the title says: “distribution of self-reported eating disorders in the sample”

Italic values: the distribution of the 84 affected respondents according to eating disorder type

Regular values: the distribution of the 84 affected respondents according to time of occurence of the eating disorders

The questionnaire contained translations of the three diagnostic subscales of EDI. Each item of EDI is measured by 6-point Likert scale (always, usually, often, sometimes, rarely, or never), scoring 0–3, where higher scores represents more severe symptoms. The diagnostic subscales are the following: Drive for Thinness subscale (7 items), Body Dissatisfaction subscale (7 items), and Bulimia subscale (9 items). The Hungarian translation of EDI was evaluated for general population by Túry et al. [26].

### Statistical analysis

We used IBM SPSS 23 statistical package. 129 questionnaires not completed properly in respect of EDI were excluded from the analysis. Questionnaires were included where the answers were given either in terms of lifetime or point prevalence. Since incomplete questionnaires were included, the number of cases in different aspects of analysis can vary.

We checked the internal consistency of the EDI subscales by Cronbach’s alpha and item-total correlations (ITC). For desirable thresholds, Garner and colleagues considered having the coefficients of internal consistency (Cronbach’s alpha) above .80 for the ED subsample, and item-scale correlation coefficients above .40. They accepted ITC below .40 in several cases they considered conceptually important. We tested whether ITC and Cronbach’s alpha values in the ED subsample met the above-mentioned criteria.

We checked the factorial validity of the three diagnostic subscales of the EDI with confirmative factor analysis (CFA; similarly to Espelage et al [22]). We compared the three-factor model to the null model and one-factor model, and initially, we assumed uncorrelated errors. Considering the significantly skewed distribution of the scales, we used maximum-likelihood estimation with bootstrap method (1000 resample). We measured the model fit with TLI and RMSEA: for the first three measures, we used the minimum threshold of .9, according to Klein [30], and for the last one, the maximum threshold of .08 according to Quintana and Maxwell [31]).

We also measured the discriminative validity of EDI by comparing scores between ED and non-ED groups on the three subscales. As the distributions of the subscales were non-normal and the group with EDs was small, we used a non-parametric test (Mann–Whitney U) for the comparison.

## Results

The internal consistency analysis showed that several items of the EDI gave inconsistent results in the analyzed sample (Table 3). To be sufficiently sensitive, the measurement has to be consistent in the group of people with EDs, and to yield specific results, the measurements must be consistent in the whole sample. We paid due heed to these considerations when deciding whether to keep or exclude an item.

**Table 3:**
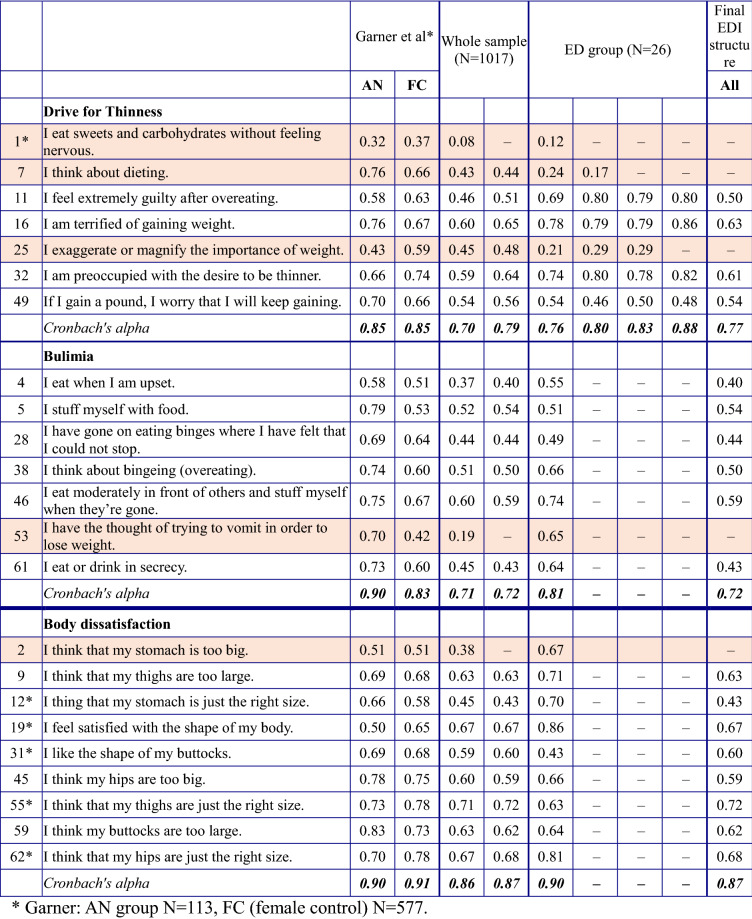
Item-total correlations of EDI items and Cronbach’s alpha values of EDI subscales

The first item on the Drive for Thinness subscale “I eat sweets and carbohydrates without feeling nervous” did not properly correspond to the subscale for either participants with EDs or the sample of pregnant women as a whole (ITC values were .12 and .08, which were considerably below the .4 threshold). Furthermore, in the case of participants with EDs the item “I think about dieting” and “I exaggerate or magnify the importance of weight” did not correlate to the subscale we intended to investigate (ITC = .17 and .29). This subscale was therefore narrowed down to four items. Cronbach’s alpha for the subscale was .77 on the whole sample and .88 on the ED subsample, and ITC values for each item were above .4. Consequently, the subscale was found to measure consistently. The exclusion of items also improved the reliability of the measurement.

On the Bulimia subscale, the item “I have the thought of trying to vomit in order to lose weight” was inconsistent in the whole pregnant sample (ITC = .19). It would have fitted properly in the ED subsample (ITC = .65), but since it measured inconsistently in the whole sample, we decided to exclude the item, reducing the Bulimia subscale to six items. Now Cronbach’s alpha was .72 on the whole and.8 in the ED subsample, and ITC values to each item reached or exceeded .4.

On the Body Dissatisfaction subscale, we excluded an item for the same reason. The statement “I think that my stomach is too big” did not match properly to the other items of the subscale in the whole sample (ITC = .38), so we left it out in spite of the fact that it would have worked well in the ED subsample (ITC = .67). Finally, the subscale was reduced to eight items and resulted in the highest Cronbach’s alpha value: .87 in the whole sample and .90 in the ED subsample. Item-total correlations were over .4 in case of every items.

The three-factor CFA model, as shown in Table 4, did not fit the data (GFI = .86; AGFI = .83; CFI = .81 RMSEA = .08). We also found that the three-factor model fitted our data much better than the null model or the one-factor model. After excluding the above five items, we fitted the three-factor CFA models again. To obtain a better fit in the case of Body Dissatisfaction, we had to allow correlation between three pairs of items (which are all semantically related to each other). Having made these changes, the fit of the model was acceptable (GFI = .92; AGFI = .89; CFI = .90; RMSEA = .07; Table 4). The factor scores are presented in Table 5, and the inter-scale correlations in Table 6.

**Table 4:** Goodness-of-fit indicators for EDI confirmatory factor analysis models

Model	GFI	AGFI	Χ^2^	Χ^2^/dfs	CFI	RMSEA
Null	.38	.31	6418.65	41.95	.00	.21
One factor	.78	.72	1888.38	14.31	.72	.12
Three factor	.86	.83	1656.41	7.30	.81	.08
Corrected three factor^a^	.92	.89	783.95	6.08	.90	.07

^a^Following items were removed, due to low internal consistency: 1, 2, 7, 25, and 53. Errors for the following items were correlated: 9 and 19; 45 and 59; 9 and 62

**Table 5:** EDI item factor loadings for confirmatory factor analysis of corrected three-factor model

Item	Factor loading (bootstrap estimation)			
	Estimate	Lower	Upper	*P*
DT				
11	.59	.49	.68	.003
16	.72	.64	.78	.003
32	.78	.72	.83	.002
49	.63	.51	.72	.003
BUL				
4	.52	.33	.67	.001
5	.65	.35	.80	.001
28	.50	.21	.70	.006
38	.58	.22	.79	.002
46	.69	.34	.85	.003
61	.55	.12	.84	.003
BODIS				
9	.79	.75	.83	.002
12*	.47	.41	.54	.001
19*	.76	.71	.79	.002
31*	.59	.54	.65	.002
45	.63	.56	.69	.002
55*	.78	.75	.81	.002
59	.65	.59	.71	.002
62*	.75	.70	.79	.001

*Items were reversed

Errors for the following items were correlated: 9 and 19; 45 and 59; 9 and 62

**Table 6:** Inter-scale correlation for confirmatory factor analysis of corrected three-factor model

Scales	Estimate	Lower	Upper	*P*
DT-BUL	.47	.31	.60	.003
DT-BODIS	.70	.63	.75	.002
BUL-BODIS	.30	.21	.39	.001

The diagnostic cut-off values for the three subscales were adjusted proportionally to adjust for the five excluded items. The proposed cut-off values are: 8 points on the Drive for Thinness subscale, 12 points on the Bulimia subscale, and 19 points on the Body Dissatisfaction subscale for the population of pregnant women.

We found a significant difference between ED and non-ED subsamples on every subscale of the EDI validated on the pregnant population (Table 7). In the absence of a clinical diagnosis, we cannot calculate exact sensitivity values, but the difference between the means indicates that subscales of the EDI (reduced by altogether five items) capture those differences in thinking and behavior that Garner and his co-researchers intended to measure.

**Table 7:** EDI mean scores in ED and non-ED groups (non-parametric Mann–Whitney *U* test)

Name of the subscale (number of current ED cases/non-ED cases)	Mean (SD)		Mann–Whitney *U* test	
	ED group	Non-ED group	*U*	*p*
EDI—Drive for thinness (23/1025)	3.26 (4.06)	0.97 (2.00)	15,703	.001
EDI—Bulimia (25/1052)	1.12 (2.15)	0.13 (0.75)	16,659	.000
EDI—Body dissatisfaction (24/1033)	9.08 (6.70)	5.40 (5.29)	16,570	.005

## Discussion

EDs can cause several prenatal, perinatal, and postnatal sequelae: among others, a higher risk for complicated course of pregnancy, miscarriage, cesarean section, poor fetal growth, postpartum depression, and breastfeeding difficulties [6, 8, 9, 32]. Since these phenomena influence the health of both mother and infant, it is essential to recognize these psychosomatic disorders as early as possible. Furthermore, because of the shame and secretiveness characteristic of this condition, and since diagnosis cannot be established before pregnancy, a valid screening tool is necessary during pregnancy. The aim of this study was to validate the EDI questionnaire in a pregnant population, using all the three diagnostic subscales of the test. As for field work, we used self-report questionnaires, which lack the stringency of a diagnostic interview but offer the benefits of anonymity. Because of their hidden character, EDs are almost certainly underreported. Anonymity increases the probability of a more realistic picture of the occurrence of symptoms, a view that is reinforced by the satisfactorily high response rate of this survey, although this must be set against the loss of diagnostic accuracy. Although the sample was not random, it was representative in an important respect: the BMI distribution of the respondents was similar to that of the female respondents of the same age in a Hungarian representative survey [33].

We surveyed 1146 women who had given birth following full-term pregnancies. The results show that certain items of the EDI function differently on a sample of pregnant women than on an average sample. To validate the EDI on a pregnant population, we needed to exclude five items—three from the Drive for Thinness subscale and one each from the Bulimia and Body Dissatisfaction subscales.

All the excluded items are clearly linked to changes in the physiological or psychological state of pregnant women, which probably accounts for these items’ limited usability. The three items removed from Drive for Thinness subscale are closely related to increased food consumption without feeling less stress during pregnancy, as found by Clark and Ogden [34]. One item (“I think about dieting”) might also be related to the increased drive towards healthy behavior observed among pregnant women. The answers to the item that was removed from the Bulimia subscale (“I have the thought of trying to vomit in order to lose weight”) might have been affected by the increase in the prevalence of nausea during pregnancy described by Lacroix et al [35]. In this case, vomiting can be caused by an ED but also can be a normal accompaniment of being pregnant. Finally, the answers to the item that was removed from the Body Dissatisfaction subscale (“I think that my stomach is too big”) could have been heavily biased by the physiological change in the shape of abdomen during pregnancy. Since the swelling of the abdomen is a natural concomitant of pregnancy, it is less likely to be perceived as a problem.

After this modification, the internal consistency of the EDI was satisfactory and it appeared to be a reliable screening tool. Cronbach’s alpha and item-total correlation values reached or exceeded the required thresholds. The differences between means on the EDI subscales showed that the test effectively measures dimensions of EDs and is thus capable of discriminating between ED and non-ED patients. However, due to the self-reporting and the fact that the reference group did not properly correspond to the target group, we could not calculate exact sensitivity and specificity.

Our results show partial similarity to the Delphi consensus study conducted among health professionals and women during or after pregnancy [15]. The Delphi consensus study showed that urges of wanting to vomit was a non-consensus item, which is similar to our finding on the inconsistency of the vomit-related item of EDI Bulimia subscale. On the other hand, distress regarding changing shape was a consensus item that is conflicting with our finding on the inconsistency of stomach size-related item of EDI Body Dissatisfaction subscale.

The results confirm that attitudes and cognitions connected to eating and attitudes to changing body weight alter during pregnancy, and these alterations have a physiological background. New forms of responsibility appear, and we can see a realignment of priorities (expecting a baby versus having an ED) [36, 37].

Changing behavior and attitudes during pregnancy improve body satisfaction and decrease the urge to control food intake. In most cases, physical symptoms of EDs moderate or disappear during pregnancy [37]. Because vomiting accompanies pregnancy, it is no longer a tool for body weight manipulation.

Earlier research has found that pregnancy can have diverse effects on ED symptoms: in some cases, pregnancy functions as a healing tool, and in others, symptom severity decreases but recurs after delivery, while in a third group, symptoms maintain during pregnancy and in the postpartum period [38].

### Limitations

The aim of our study was to evaluate EDI among pregnant women. Data were collected after delivery, during the first 3 days of the postpartum period. To adjust data, measurement was based on retrospective questions. The fact that our results showed such kind of deviations from the non-specific population that can be linked to changes in the physiological or psychological state of pregnant women support the validity of our results. Further research should confirm our results based on a survey among pregnant women with non-retrospective question.

## Conclusion

The findings of our research show that the measurability of signs and symptoms of EDs change during pregnancy in parallel with physiological and psychological changes. The validity of well-established and widely used diagnostic tools such as EDI also change. The instrument therefore requires modification. The possible consequences of hidden ED for pregnancy—in addition to its importance in eating disorder therapy—have considerable relevance for prenatal and postnatal care.

What is already known on this subject?

EDs connected with pregnancy have a special importance, because the onset of the EDs occurs at a critical phase of women’s reproductive life, psychological factors make diagnosis more difficult in case of pregnancy, and EDs can have numerous consequences for pregnancy. The EDI is one of the most widely used instruments for the assessment of EDs, which was developed and validated by Garner and colleagues. Physiological changes and events of the pregnancy can cause problems in screening pregnant women for EDs.

What your study adds?

Three diagnostic subscales of EDI were modified and evaluated for pregnant population. The evaluated tool can be used in screening hidden EDs among pregnant women.

## Data Availability

The datasets generated and analyzed during the study are currently not publicly available but are available from the corresponding author on reasonable request.
